# AI-enhanced clinical trial-in-a-dish platform for improved DILI risk classification and mechanistic insights into hepatotoxicity

**DOI:** 10.3389/ftox.2026.1740791

**Published:** 2026-03-13

**Authors:** Sara Cherradi, Salomé Roux, Caroline Bailleux, Clément Devic, Colin Debaigt, Kamelia Guerda, Samantha Luciano, Mélanie Dalle, Hong Tuan Duong

**Affiliations:** 1 PredictCan Biotechnologies SAS, Biopôle Euromédecine, Grabels, France; 2 Department of Oncology, Centre Antoine LACASSAGNE, Nice, France; 3 Département d'Épidémiologie, de Biostatistique et des Données de Santé (DEBDS), Centre Antoine LACASSAGNE, Nice, France; 4 Délégation à la Recherche Clinique et à l’Innovation (DRCI), Centre Antoine LACASSAGNE, Nice, France; 5 Centre de Ressources Biologiques, Centre Antoine LACASSAGNE, Nice, France

**Keywords:** artificial intelligence, drug-induced liver injury, mechanism-based toxicity, microphysiological systems, predictive toxicology, risk classification

## Abstract

Drug-induced liver injury (DILI) remains a leading cause of clinical trial attrition and post-marketing drug withdrawals. Its prediction is hindered by the limited physiological relevance and interindividual variability captured in conventional preclinical models. To overcome this, we developed a human serum-derived educated spheroid system incorporating human blood sera from donors to generate liver spheroids that recapitulate human hepatic diversity. This platform enables clinical trial-in-a-dish studies and supports acute and chronic treatment regimens. Using a panel of drugs spanning the full DILI risk spectrum, we evaluated hepatotoxic potential through a proprietary AI-driven algorithm that integrates severity and incidence metrics at therapeutic concentrations. Our platform reliably distinguished low-risk from high-risk DILI compounds and recapitulated both dose-dependent and idiosyncratic toxicity profiles. Notably, ximelagatran-induced DILI was only detected under chronic exposure conditions, mirroring clinical outcomes. Transcriptomic profiling revealed innate immune activation in DILI-positive individuals. STRING analysis further implicated HLA-DRB1 and HLA-DQA1 interactions via *VIM* upregulation in macrophages and dendritic cells, suggesting a mechanistic link to immune-mediated iDILI. In exploratory prospective studies, our system predicted ribociclib-induced grade 3 DILI in one ER+/HER2− breast cancer patient and absence of DILI in two patients, consistent with clinical outcomes. These findings highlight the value of integrating our model with our AI-based mapping strategy to enable mechanistic classification of DILI, deconvolution of immune-related toxicity, and prediction of patient-specific risk. Our platform represents a step toward personalized hepatotoxicity assessment and improved translational toxicology strategies.

## Introduction

Drug-induced liver injury (DILI) is a significant challenge for pharmaceutical companies during drug development and is one of the leading causes of drug attrition during clinical trials and post-marketing surveillance (Norman, 2020). This phenomenon is categorized from mild, reversible liver enzyme elevations to severe, potentially fatal liver failure (Bjornsson and Bjornsson, 2022). DILI is usually classified into intrinsic type, where liver injury is dose-dependent, predictable, and injury may resolve spontaneously, or into idiosyncratic type, where it is unpredictable, dose-independent, and does not always resolve spontaneously (Hosack et al., 2023). Importantly, a drug can have dual characteristics exhibiting both intrinsic and idiosyncratic toxicity making its prediction challenging in a population (Roth and Ganey, 2010; Yokoi and Oda, 2021). Indeed, some drugs can cause intrinsic DILI in the general population via direct hepatotoxic effects, but in a subset of patients, they may also provoke idiosyncratic DILI through complex immune-mediated reactions. Thus, DILI is difficult to detect during preclinical and early clinical trials.

Preclinical models such as cell lines, primary human hepatocytes (PHHs), human liver microtissues, or animal models, while useful, can sometimes misclassify compounds with a lower risk of causing DILI (less-DILI compounds) as high-risk (DILI positive) inducing premature termination of the development of otherwise promising drugs (Walker et al., 2020). Donepezil is an example of false positive inconsistently classified by human liver microtissues (Walker et al., 2020). On the other hand, these preclinical models can also fail to detect hepatotoxicity, which later manifests in humans causing serious consequences, including the removal of drugs from the market after they have been approved and used by patients. An example of false negative is sitaxentan, an endothelin receptor antagonist used to treat pulmonary arterial hypertension, that was withdrawn from the market due to severe liver toxicity observed in some patients in both acute and chronic treatment, despite it was considered as safe by preclinical studies (Lee et al., 2011; Owen et al., 2012). The mechanism of sitaxentan-induced idiosyncratic DILI is so far poorly understood (Lee et al., 2011). Ximelagatran, an oral direct thrombin inhibitor, is another example of a drug that was withdrawn due to liver toxicity that was not detected in preclinical studies. Short-term use (<12 days) in humans including the phase 3 clinical trials did not show any hepatotoxicity. However, long-term (>35 days) use of ximelagatran revealed a higher frequency of elevated hepatic enzyme levels (>3x upper limit of normal plasma ALT) with an incidence of 7.9% (Keisu and Andersson, 2010). The termination of the ximelagatran development was triggered by safety data from a 35-day study that showed that severe hepatic injury could occur in a patient after the exposure to the drug has been completed, and that regular liver function monitoring did not help to mitigate the possible risk of severe hepatic injury. Although extensively tested during its development, no standard preclinical toxicological studies have revealed an indication that ximelagatran can cause hepatic injury (Keisu and Andersson, 2010). Importantly, studies using human primary liver cell-based *in vitro* models were not able to define mechanisms explaining the liver injury observed in long-term clinical trials suggesting that ximelagatran-mediated idiosyncratic DILI is presumably multifactorial and individual-dependent. These observations clearly highlight the critical need for more accurate and predictive microphysiological systems (MPS) that better mimic the human liver environment to eliminate false positive and false negative DILI as early as possible in the drug development process. This task is difficult to achieve at preclinical stage because both negative and positive DILI drugs can cause idiosyncratic hepatotoxicity in some people (Chen et al., 2011). It is believed that this can happen due to various factors, including genetic predisposition, underlying health conditions, lifestyle, and interactions with other medications. Moreover, blood systemic components play also a crucial role in the interindividual variability of DILI because of the subject-specific composition of metabolites, cytokines and immune mediators, proteins and enzymes, and microbiome-derived metabolites. Indeed, blood serum represents 40%–50% (v/v) of the whole blood and contains diverse factors including hormones, cytokines, extracellular vesicles, and gut microbiota-derived peptidoglycan fragments, that are expressed at different levels and in an individual-dependent manner (Kleiner et al., 2013; Arapidi et al., 2024). There is evidence that microbes can impact the distant organ in a contact-independent manner via the gut microbiota-derived peptidoglycan fragments from the cell wall, that enter the blood stream. Host organs differentially take up circulating peptidoglycan fragments (Schroeder and Backhed, 2016). In the liver, it has been shown experimentally that the maximal uptake was 1 h and then remained stable for at least 6 h (Wheeler et al., 2023). This systemic diversity of the serum’s composition may predispose certain subjects to DILI risk. Thus, to mitigate false positive and false negative DILI, MPS should represent human diversity as everyone may have predisposition to DILI regardless of whether the drug in question is classified as a less-DILI or most-DILI compound.

Presently, preclinical models analyze DILI risk of a drug in binary mode. These studies are generally performed at concentrations much higher than clinical doses (i.e., up to 100x C_max_) and considered a compound as DILI positive if cell death occurred before 100x C_max_. This approach can be an issue as the mechanism of toxicity may differ at lower and more physiologically relevant concentrations. Moreover, this binary analysis does not highlight the idiosyncratic features of DILI. Idiosyncratic DILI (iDILI) often occurs at doses well tolerated by most people and depends on individual susceptibilities (Uetrecht, 2019). To date, no genetic, nor other factors have been found to reliably predict iDILI. Thus, it is a hard task to predict its occurrence with standard preclinical approaches.

Clinical trial-in-a-dish approach is particularly useful for assessing interindividual variability in DILI. Thus, it is an excellent strategy to monitor early enough iDILI at preclinical stage. Nevertheless, running a clinical trial-in-a-dish to capture iDILI is supposed to have a system that reproduces interindividual biodiversity. We have previously developed an individual-centric spheroid system using blood sera to better mimic the human liver’s response to drugs than standard preclinical models, reproducing interindividual variability (Cherradi et al., 2023; Roux et al., 2024b; Roux et al., 2024a). In the present work, we use this technology in a clinical trial-in-a-dish platform to mitigate both false positive and false negative DILI de-risking drug development. Generally, to effectively assess interindividual variability and ensure statistically significant results, it is recommended to use cells derived from at least 20 different individuals to have a robust clinical trial-in-a-dish (Martinez-Mesa et al., 2014; Althubaiti, 2023). The primary objective of phase 1 studies is to assess the safety of a drug candidate, and these trials are usually conducted in 50–80 healthy volunteers. Therefore, we generated large cohorts of individual-centric spheroids from 50 to 80 healthy individuals to analyze iDILI of a panel of 9 drugs including donepezil, a less-DILI drug that was wrongly flagged as DILI positive, and of sitaxentan and ximelagatran, two most-DILI drugs classified with no indication of hepatotoxicity by other preclinical models. We used a proprietary AI-powered algorithm and showed that our *in vitro* system could correctly categorize less-DILI and most-DILI concern drugs based on severity and incidence metrics. We exploited the flexibility of our technology to uncover the mechanism of ximelagatran-mediated idiosyncratic liver toxicity. Finally, we demonstrated in exploratory prospective case studies that our human serum-derived educated spheroid system was able to predict with a good accuracy ribociclib-mediated DILI in breast cancer patients validating the robustness of our technology and paving the way for development of a diagnostic companion test and personalized medicine.

## Materials and methods

### Biological samples, cell lines, and reagents

Patient’s blood was collected and processed according to the protocol described elsewhere (Cherradi et al., 2023). Blood samples from healthy donors were obtained from the Etablissement Français du Sang (EFS) Hauts de France–Normandie. The research protocol was conducted under French legal guidelines and fulfilled the requirements of the local institutional ethics committee. For breast cancer patients, all blood samples were collected after the patients were informed and have provided written consent, within the framework of the BEST program biobank at the Centre Antoine LACASSAGNE for research with potential clinical applications, and in accordance with CNIL MR003 regulations regarding the use of their data. The trial is registered on ClinicalTrials.gov (ID: NCT06851975). This study has been approved by Comité de Protection des Personnes Sud-Méditerranée II (CPP: 219C17). Patient biological samples are identified by CODECOH n°AC-2020-3933. Patient follow-up was performed as part of routine care and liver toxicities were obtained from medical records in accordance with applicable procedures. The sample were then managed by the Centre de Ressources Biologiques, handled and stored in accordance with the study protocol. All processes were carried out in compliance with the requirements of ISO-20387, the international standards for biobanking. The study was approved by the “Direction Générale de la recherche et de l’innovation” (CODECOH, n°DC-2021–4779). This project does not involve the human person according to the legislation (article L1121-1 du code de la santé publique).

Lidocaine, streptomycin, donepezil, verapamil, imipramine, sitaxentan, ximelagatran, APAP (N-acetyl-p-aminophenol) also named “paracetamol”, and ribociclib were purchased from TargetMol (Linz, Austria).

Hepatocyte (HepG2) and monocyte (THP-1) lines were from ATCC (Molsheim, France). Hepatic stellate cell line (TWNT-1) was from Glow Biologics (Tarrytown, NY, USA). Cell culture reagents were provided by StemCell (Saint Égrève, France). Hepatocytes, monocytes, and hepatic stellate cells were conditioned for a minimum of 2 weeks in hSELS medium (PredictCan Biotechnologies, Grabels, France) before use to sensitize them to cell educating technology. Human serum-derived educated spheroids were generated and cultured in hSELS medium supplemented with depleted human serum prepared according to our proprietary in-house protocol. Specifically, human serum-derived educated spheroids were established in 384-well ultra-low attachment plates by seeding 90,000 HepG2 cells, 10,000 TWNT-1 cells, and 1,000 THP-1 cells per well. Spheroids were allowed to form over 3 days in hSELS medium supplemented with 50% human serum, which was maintained throughout the entire experiment to preserve the physiological environment. Human plasma was initially evaluated in the culture system. However, it induced significant baseline cytotoxicity independent of drug treatment, limiting interpretation of drug responses. Human serum was prepared from whole blood following standard clotting procedures and used at 50% in all experiments as it was substantially better tolerated in our system.

### Treatment protocols

For acute treatment, individual-centric spheroids were exposed for 3 days to drugs at concentrations ranging from 0.01x to 100x C_max_. For chronic treatment, individual-centric spheroids were exposed to drugs up to 16 days with redosing at day 7 and day 14.

All drugs were prepared as DMSO stock solutions. The final DMSO concentration in culture medium did not exceed 2% (v/v). Control experiments confirmed that this concentration does not induce cytotoxicity in our system.

A transwell co-culture system was also employed, in which individual-centric spheroids were cultured in the bottom chamber, while monocyte-derived macrophages and dendritic cells, prepared with the same batch of blood serum, were seeded in the upper transwell insert (#3391, Corning). This configuration allowed for paracrine signaling and soluble factor exchange.

### Cell viability, metabolic activity, and Ki67

Cell viability, metabolic activity, and Ki67 were measured using CellTiterGlo or Lumit® hKi-67 Immunoassay (Promega, Charbonnières-les-Bains, France) according to the manufacturer’s instructions.

### Quantitative PCR

RNA extraction was performed using RNeasy Mini Kit (QIAGEN) according to manufacturer’s instructions. Reverse transcription was performed using OneScript® RT Mix for qPCR w/gDNAOut (Ozyme) followed by an amplification with ONEGreen® FAST qPCR Premix (Ozyme) according to manufacturer’s instructions. Primers (CYP1A1, CYP1A2, CYP2B6, CYP2C9, CYP2C19, CYP2D6, CYP3A4, CYP3A5, CYP3A7, HNF1A, HNF4A, CEBPA, CEBPB, GATA2, GATA4, FOXA3, HK2, LDHA, PKM2, SIRT1, PGC1α, COX1, and RRP36) were purchased from BIORAD. Quantitative PCR was performed on an IVDR-compliant thermal cycler (Thermo Fisher Scientific, QuantStudio 5 Dx Real-Time PCR System). All CTs were collected and the ΔCTs were calculated by subtracting to RRP36 (housekeeping gene) CT. Relative expression to GusB for each gene was calculated by using the formula RE = 2^−ΔCT^.

### RNA sequencing

RNA extraction was performed using RNeasy Mini Kit (QIAGEN) according to manufacturer’s instructions and then stored at −80 °C prior shipping to QIAGEN sequencing service (Germany). The RNA-seq datasets used in this study are the exclusive property of PredictCan Biotechnologies and are subject to confidentiality and data protection agreements. As such, these data will not be shared or made publicly available.

### Data processing and AI-powered algorithm analysis

Cell viability data were processed in GraphPad Prism v9 (Dotmatics, San Diego, CA) to generate LogIC_50_ and HillSlope values for each individual, which were subsequently input into a proprietary AI-powered algorithm to generate non-linear regression curves showing dose-dependent and dose-independent toxicity, to identify subjects at risk for DILI, and to determine severity and incidence metrics for each drug tested.

## Results

### HepG2 line acquires a normal hepatocyte-like phenotype under specific cell culture conditions with a human serum-based medium

**FIGURE 1:**
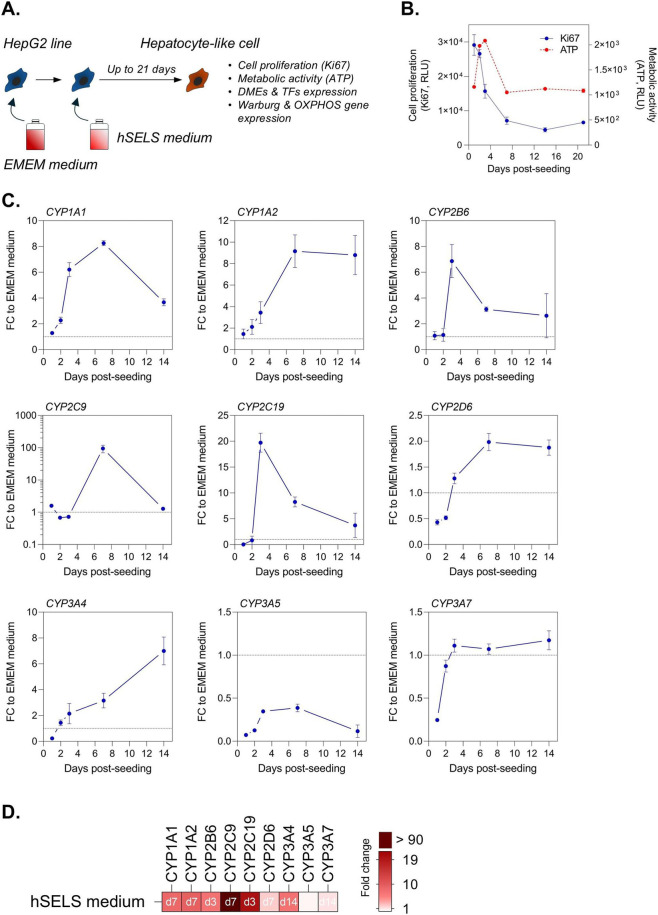
Reversion of HepG2 line to normal-like hepatocytes in hSELS medium. **(A)** Workflow. **(B)** Longitudinal assessment of cell growth and metabolic activity. Ki67 and ATP measurements were performed on HepG2 cultured in hSELS medium up to 21 days. Results, relative luminescence unit (RLU), are expressed as mean ± s. e.m. Technical triplicates were done for each time point. **(C)** Time-resolved monitoring of expression of major CYP family members. Expression of CYPs was performed by quantitative PCR. Results are expressed as mean ± s. e.m. fold change (FC) to standard EMEM medium cell culture condition. Technical triplicates were done for each time point. **(D)** Representation of the optimal time point for peak CYP expression. Heatmap showing the maximal FC to standard EMEM medium cell culture condition and the optimal time point.

**FIGURE 2:**
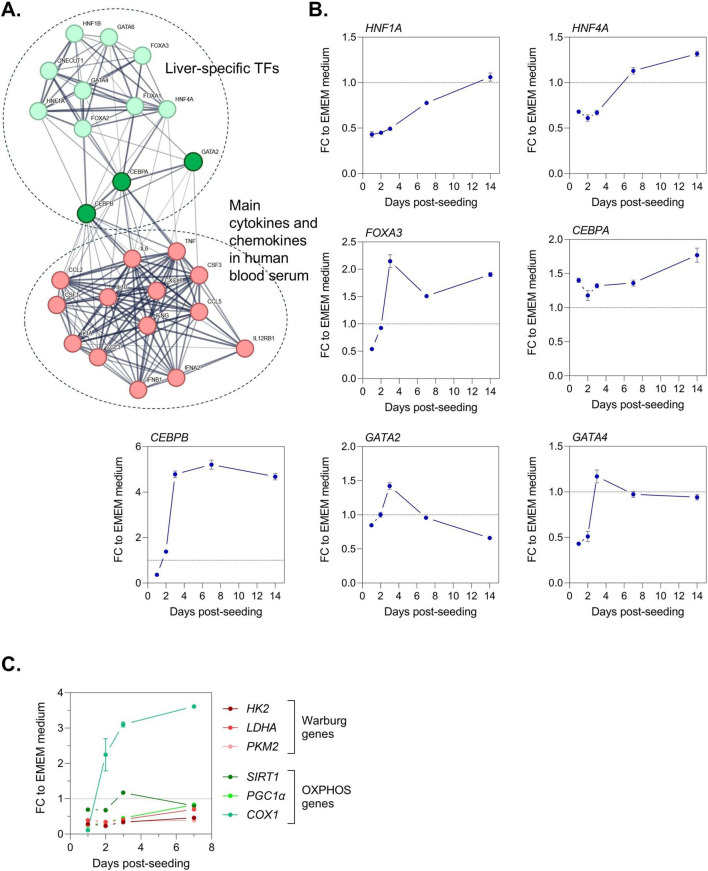
Deciphering liver-specific transcription factor networks in hepatic cancer cell line reprogramming. **(A)** STRING analysis identifying CEBPA, CEBPB, and GATA2 as principal nodes connecting liver-specific transcription factors to main cytokines and chemokines found in human blood serum. **(B)** Temporal monitoring of liver-specific transcription factors. Expression levels were measured by quantitative PCR. Results are expressed as mean ± s. e.m. fold change (FC) to standard EMEM medium cell culture condition. Technical triplicates were done for each time point. **(C)** Time-dependent analysis of Warburg and OXPHOS gene expression in cellular energy metabolism. Expression levels were measured by quantitative PCR. Results are expressed as mean ± s. e.m. fold change (FC) to standard EMEM medium cell culture condition. Technical triplicates were done for each time point.

Presently, PHHs are widely considered the gold standard for assessing DILI in preclinical studies. However, the collection of human liver tissues to isolate PHHs presents significant limitations for generating reliable and diverse *in vitro* models due to limited tissue availability making that once a donor’s PHHs are used, they cannot be replenished, and inadequate representation of human biodiversity because of a small number of donors. To overcome these issues, we developed a human serum-derived educated spheroid system combining cell lines such as HepG2 or TWNT-1, and human serum to mimic individually primary liver cells phenotype reproducing human biodiversity. We have selected HepG2 cells because they are convenient and inexpensive despite their lower metabolic competency compared to primary human hepatocytes (Cherradi et al., 2023; Roux et al., 2024b; Roux et al., 2024a). HepG2 line cultured in our proprietary human serum-based hSELS medium acquired hepatocyte-like cell characteristics with a reduction of cell proliferation (Figures 1A,B). We observed that the overall metabolic activity was not compromised (Figure 1B) and an increase in expression of major CYP family members (Figure 1C) with the highest expression at day 3 for CYP2B6 and CYP2C19, at day 7 for CYP1A1, CYP1A2, CYP2C19, and CYP2D6, and at day 14 for CYP3A4 and CYP3A7 (Figure 1D). To understand the mechanism behind the reversion of hepatic cancer cell line towards hepatocyte-like cell phenotype, we performed a STRING analysis highlighting CEBPA, CEBPB, and GATA2 as principal nodes connecting liver-specific transcription factors to main cytokines and chemokines found in human blood serum (Figure 2A). As expected, we found that *CEBPA*, *CEBPB*, *GATA2*, *FOXA3*, and *HNF4A* expressions were increased in HepG2 cultured in hSELS medium as compared to standard cell culture condition with EMEM medium (Figure 2B). Consequently, we observed an important metabolic shift that reflects a reversion of cancer cells toward a more normal, differentiated phenotype with a reduction in the expression of Warburg genes and an upregulation of OXPHOS genes indicating a return to normal energy production through oxidative phosphorylation (Figure 2C). Overall, our data indicate that HepG2 cell line cultured in hSELS medium exhibits features consistent with a shift toward a more normal-like hepatocyte phenotype.

### Individual-centric spheroids did not show cell over proliferation in long-term cell culture conditions

**FIGURE 3:**
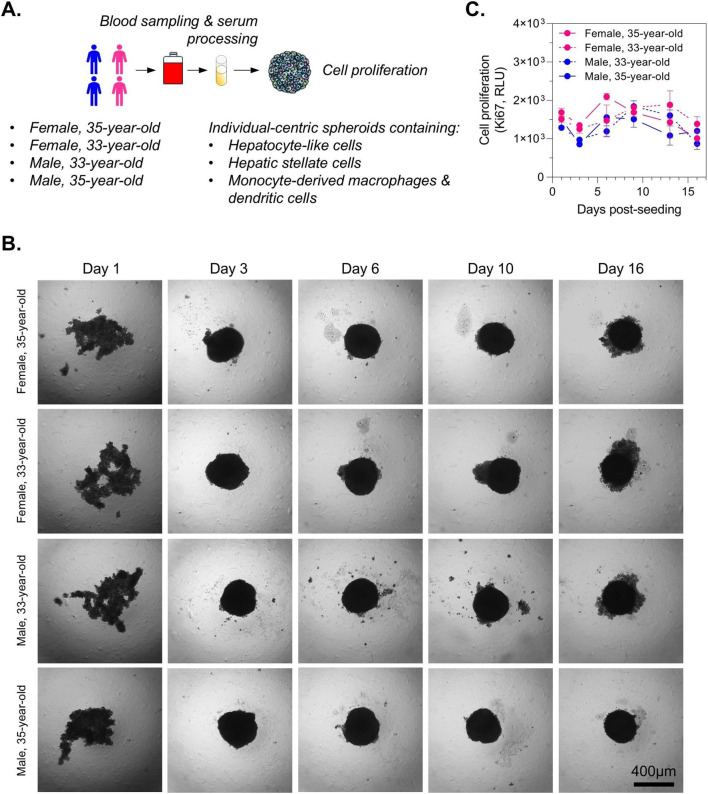
Absence of overproliferation in individual-centric spheroids maintained under long-term culture conditions. **(A)** Workflow. **(B)** Light microscopy imaging of individual-centric spheroids. Multicellular individual-centric spheroids were generated using blood sera from 4 individuals. Scale bar represents 400 µm. **(C)** Longitudinal assessment of cell growth. Ki67 measurements were performed up to 16 days post-seeding. Results, relative luminescence unit (RLU), are expressed as mean ± s. e.m. Technical triplicates were done for each time point.

The use of immortalized cell lines such as HepG2 or Huh7 to generate spheroids for DILI assessment often suffers from excessive uncontrolled cell proliferation resulting in enlarged spheroids with a necrotic core that can alter drug penetration and drug responses and thereby reducing the physiological relevance and predictive accuracy of the model. To verify that our individual-centric spheroids, that contain HepG2-derived hepatocyte-like cells, hepatic stellate cells and monocyte-derived macrophages and dendritic cells, do not overgrowth, we generated spheroids using blood sera from 2 females and 2 males and then monitored cell proliferation by measuring Ki67 up to 16 days post-seeding (Figure 3A). As expected, we found that spheroids were compacted already on day 3 and no enlarged spheroids were observed up to day 16 (Figure 3B). Moreover, we measured a stable Ki67 expression from day 1 to day 16 suggesting an absence of uncontrolled proliferating state (Figure 3C). Our data confirmed that individual-centric spheroids can be used for chronic treatments up to 16 days without altering individual’s responses to drugs.

### Individual-centric spheroids showed individual’s responses to drugs and high reproducibility across independent experiments

**FIGURE 4:**
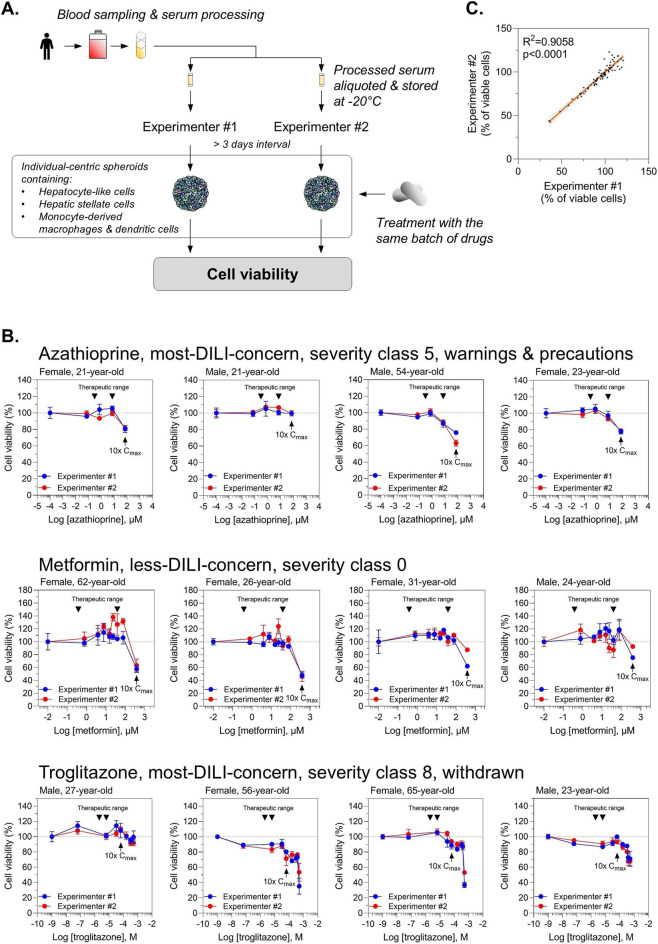
Reproducible performance across independent experimental conditions and users. **(A)** Workflow. **(B)** Data consistency across two users and experiments highlighting reproducibility and robustness of the model. Individual-centric spheroids were treated for 3 days with azathioprine, metformin, or troglitazone, and cell viability measured using CellTiterGlo. Results are expressed as percentage of viable cells ±s. e.m. Technical triplicates were done for each time point. Triangle marks indicate therapeutic range for each drug. **(C)** Evaluating reproducibility through correlation of data from independent experiments and users. A linear correlation curve was generated using data from 80 treatment conditions, 3 drugs, and 2 independent experiments performed by 2 users.

Clinical trial-in-a-dish is the best strategy to assess people’s biodiversity in drug-mediated liver toxicity identifying subjects at risk for iDILI. However, to ensure the reliability of clinical trial-in-a-dish approaches, the *in vitro* model used must capture interindividual variability in drug responses and exhibit high reproducibility across independent experiments and different operators. We collected blood from different donors, prepared and processed sera, aliquoted them, and then stored them at −20 °C until use. These sera were used to generate individual-centric spheroids by 2 experimenters in 2 independent experiments with more than 3 days interval (Figure 4A). Obtained individual-centric spheroids were treated with the same batch of drugs to analyze liver toxicity. We selected metformin, a biguanide and non-hepatotoxicant with lactic acidosis as primary safety concern, azathioprine, a purine analog, and troglitazone, a thiazolidinedione, both most-DILI concern drugs, for this reproducibility and interindividual variability study. As expected, we found a heterogeneity in drug-mediated cell death regardless of drug class. Out of 4 subjects tested, we did not find any cell death within therapeutic range for metformin, while a reduction in cell viability was observed in 1 out of 4 treated with azathioprine and 2 out of 4 with troglitazone (Figure 4B). Importantly, we demonstrated that our results were highly reproducible across experimenters (R^2^ = 0.9058, p < 0.0001) regardless of drug class validating our human serum-derived educated spheroid system for clinical trial-in-a-dish approaches (Figures 4B,C). Moreover, since frozen serum can be used with comparable efficacy to fresh serum (data not shown), individual-centric spheroids can be generated and analyzed in different laboratories using the same donor-derived batch under identical experimental conditions and drug treatments, to demonstrate the reproducibility and transferability of the system.

### Individual-centric spheroids identified subjects with dose-dependent and dose-independent toxicity at clinically relevant drug exposure

**FIGURE 5:**
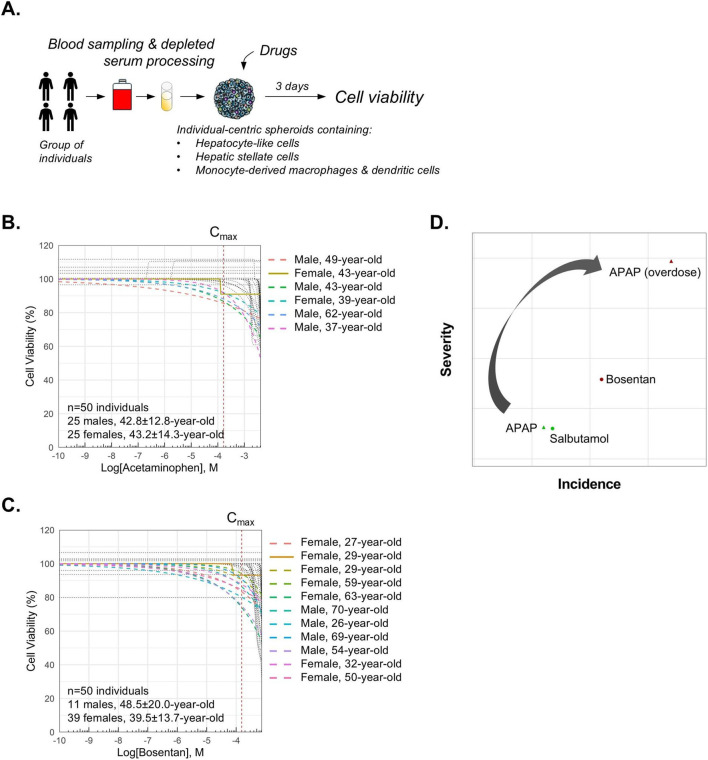
Monitoring dose-dependent hepatotoxicity. **(A)** Workflow. **(B**,**C)** Establishment of a cohort of 50 individual-centric spheroids, each generated from the blood serum of a unique donor. Individual-centric spheroids were then treated (9 doses and control) with acetaminophen or with bosentan for 3 days. Cell viability was measured using CellTiterGlo. Non-linear regression curves are shown for each individual. Technical triplicates were done for each dose. **(D)** A machine learning–driven approach to assess hepatotoxicity risk at overdose using dual risk metrics. Overdose condition was emulated by setting the dose to 25x C_max_. Scatter plot shows liver toxicity risk at therapeutic dose and at overdose for APAP and bosentan.

Intrinsic DILI is generally not a major concern for the pharmaceutical industry because it is dose-dependent, predictable across populations, and its mechanisms are typically well understood. To assess whether our *in vitro* system can monitor dose-dependent toxicity, we analyzed in a cohort of 50 individuals the effect of acetaminophen (APAP), a prototypical intrinsic DILI drug that causes predictable and dose-related toxicity in nearly all individuals at high concentrations(Jaeschke and Ramachandran, 2024), and of bosentan, a drug that causes dose-dependent and reversible liver injury in 2%–18% of patients (Fattinger et al., 2001) (Figure 5A). We found that APAP induced a dose-dependent toxicity before C_max_ in 5 subjects, a 49-year-old male, a 43-year-old male, a 39-year-old female, a 62-year-old male, and a 37-year-old male (Figure 5B). The case of bosentan-induced liver injury is somewhat ambiguous and occupies a gray area within the DILI classification framework. Indeed, while bosentan is generally considered to cause idiosyncratic DILI i.e., characterized by rare instances of severe liver injury and potential immune-mediated mechanisms, it also exhibits features typical of intrinsic DILI. Notably, bosentan has been associated with dose-dependent, cholestatic liver injury in up to 12% of patients (Fattinger et al., 2001). Our results from a cohort of 50 individuals, clearly showed that bosentan induced a dose-dependent cell death in 10 subjects out of 50 (20%), and a dose-independent toxicity in a 29-year-old female (Figure 5C). Using a proprietary machine learning–based algorithm that incorporates both severity and incidence metrics at therapeutic dose, i.e., C_max_, to evaluate DILI risk, we emulated DILI risk at overdose of APAP, i.e., at 25x C_max_. We found a clear shift of incidence and of severity confirming that APAP has a low hepatotoxicity profile at therapeutic dose but then it is highly toxic for the liver at overdoses (Figure 5D). To contextualize the positioning of APAP and bosentan on the scatter plot, salbutamol, a well-characterized no-DILI compound was included as a negative control. Importantly, the data for this compound was generated in a previous study using the same experimental platform, and the incidence and severity metrics were determined using the same machine learning-based algorithm applied in the current analysis. This benchmarking allowed for a more accurate interpretation of the data, confirming that APAP and bosentan are associated with liver toxicity. Taken together, our data demonstrated that our *in vitro* system could capture dose-dependent liver toxicity.

**FIGURE 6:**
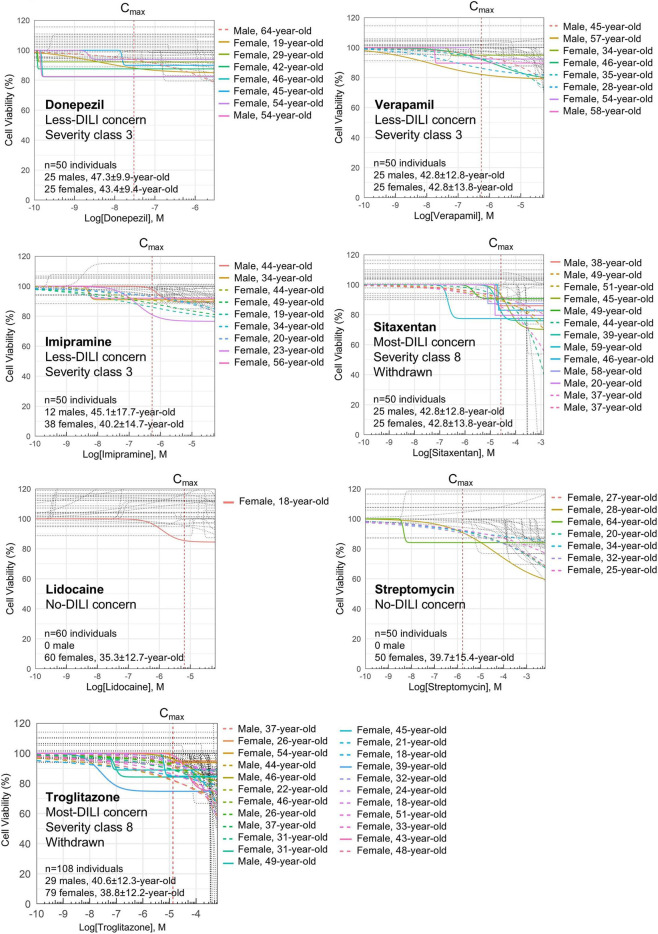
Monitoring dose-dependent and dose-independent hepatotoxicity of idiosyncratic DILI-associated drugs. A cohort of at least 50 personalized spheroids created from the blood sera of human donors, was generated and then treated with donepezil, verapamil, imipramine, or sitaxentan. Lidocaine and streptomycin are 2 no-DILI drugs and were used as control to confirm the absence of hepatotoxicity. Cell viability was measured using CellTiterGlo. Non-linear regression curves are shown for each individual. Technical triplicates were done for each dose.

**FIGURE 7:**
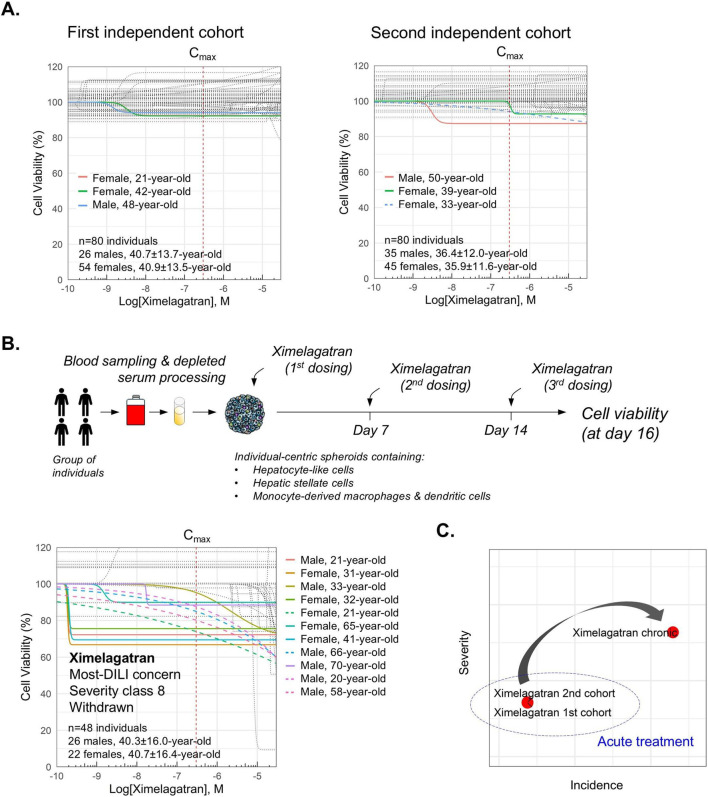
Analysis of ximelagatran-mediated DILI upon acute and chronic exposure at therapeutic dose. **(A)** Cohorts of 80 individual-centric spheroids, each generated from the blood serum of a unique donor, were built to assess ximelagatran-induced DILI upon 3 days of exposure in 2 independent experiments. Cell viability was measured using CellTiterGlo. Non-linear regression curves are shown for each individual. Technical triplicates were done for each dose. **(B)** Workflow and results showing ximelagatran-mediated DILI upon chronic treatment with repeated dosing. A cohort of 48 individual-centric spheroids, each generated from the blood serum of a unique donor, were created for this study. Cell viability was measured on day 16 post-treatment using CellTiterGlo. Non-linear regression curves are shown for each individual. Technical triplicates were done for each dose. **(C)** Scatter plot shows liver toxicity of ximelagatran upon acute and chronic exposures.

**FIGURE 8:**
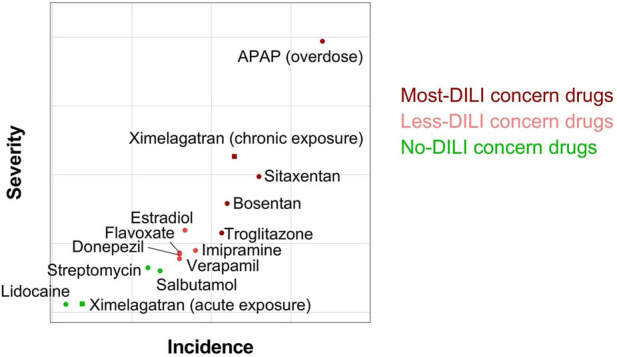
Classification of DILI-associated drugs with proprietary AI-powered dual metrics algorithm. Scatter plot shows the incidence and the severity of liver toxicity of lidocaine, streptomycin, salbutamol, flavoxate, donepezil, verapamil, imipramine, estradiol, troglitazone, bosentan, sitaxentan, APAP, and ximelagatran.

Unlike dose-dependent hepatotoxicity, idiosyncratic DILI is difficult to predict, can occur at clinically relevant doses, and does not correlate with the administered dose, posing significant barriers to drug development and regulatory approval. We analyzed six drugs donepezil, verapamil, imipramine, sitaxentan, troglitazone, and ximelagatran, that are known to cause idiosyncratic DILI, and two drugs, lidocaine and streptomycin, categorized by the FDA as no-DILI compounds, on a total number of 626 individuals including males and females with an age range from 18 to 70-year-old. As expected, these drugs showed more cases of dose-independent toxicity than APAP or bosentan, aligning with their classification as idiosyncratic DILI compounds. (Figures 6, 7). Interestingly, we observed that ximelagatran did not exhibit significant liver toxicity during acute exposure in two independent cohorts of 80 individuals each (Figure 7A). However, upon chronic administration, increased hepatotoxicity emerged (Figure 7B), aligning with clinical observations where liver injury associated with ximelagatran typically appeared after more than 35 days of use. Our findings recapitulate this delayed toxicity profile, underscoring the importance of long-term monitoring (Figure 7C). Next, data from these drugs were processed through our proprietary machine learning pipeline to generate compound-specific severity and incidence metrics. Moreover, to enhance the analytical framework, we incorporated legacy data derived from prior pharmacological studies conducted on the same platform. These datasets included validated incidence and severity metrics related to DILI, which were used to inform model development and performance evaluation. Notably, the dataset included salbutamol, classified as a no-DILI-concern drug, and estradiol and flavoxate, which are considered to have a lower DILI risk. A scatter plot based on the incidence and severity metrics was subsequently generated to enable a clearer visualization of drug classification patterns, particularly in distinguishing compounds across the DILI risk spectrum. As expected, lidocaine, salbutamol, and streptomycin were categorized as non-hepatotoxic in our analysis. In contrast, donepezil, flavoxate, verapamil, estradiol, and imipramine displayed evidence of DILI liability, albeit at lower levels compared to sitaxentan, troglitazone, APAP, and ximelagatran (Figure 8). These results are consistent with the FDA’s classification, which places the former group in the less-DILI concern category and the latter in the most-DILI concern category. The current work therefore builds on a broader foundation of experimental validation while introducing a novel analytical approach.

### Analyzing ximelagatran-mediated immune response in DILI positive and DILI negative subjects

**FIGURE 9:**
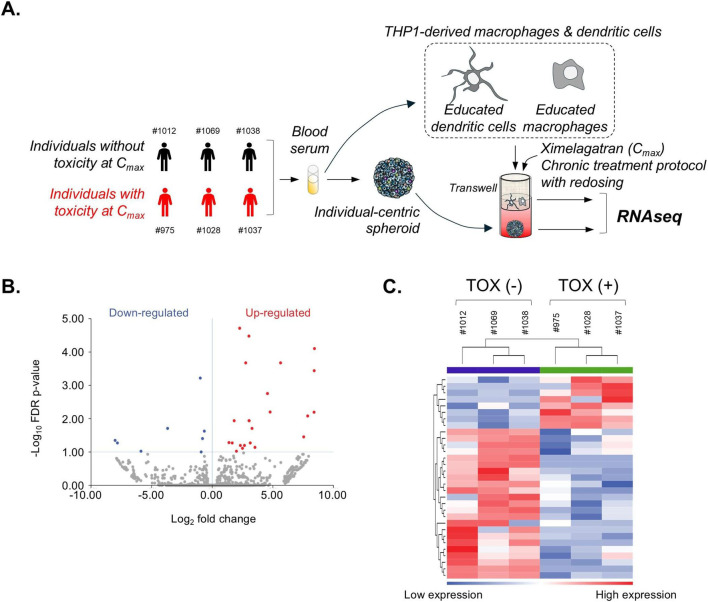
Uncovering mechanisms of ximelagatran-mediated DILI through transcriptomic profiling of liver spheroids from individuals with defined DILI risk profiles. **(A)** Workflow. **(B)** Differential gene expression volcano plot comparing TOX-positive and TOX-negative liver spheroids. **(C)** Hierarchical clustering heatmap illustrating gene expression differences in liver spheroids from three DILI-risk and three non-risk individuals.

**FIGURE 10:**
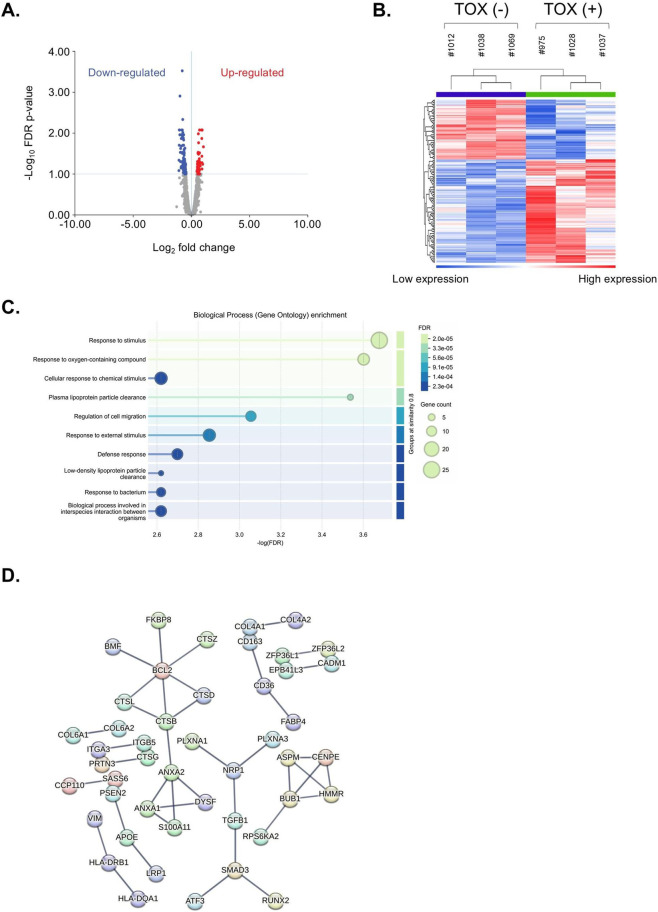
Analysis of ximelagatran-mediated hepatotoxicity in macrophages and dendritic cells of the innate immune system **(A)** Volcano plot displaying differentially expressed genes in monocyte-derived macrophages and dendritic cells under TOX (+) versus TOX (−) conditions. **(B)** Hierarchical clustering of 172 differentially expressed genes in monocyte-derived macrophages and dendritic cells derived from individuals with (n = 3) and without (n = 3) DILI risk. **(C)** GO enrichment analysis of the 27 genes upregulated in the TOX (+) condition, with known associations to DILI-related pathways or mechanisms. **(D)** STRING analysis was performed to explore potential interactions between genes upregulated in the TOX (+) condition in monocyte-derived macrophages and dendritic cells and the HLA class II genes HLA-DRB1 and HLA-DQA1.

In idiosyncratic DILI, the innate immune response, including activation of macrophages and dendritic cells, typically precedes and facilitates the adaptive immune response, thereby amplifying liver toxicity. Thus, to decipher the mechanism of ximelagatran-mediated DILI, we generated individual-centric spheroids and monocyte-derived macrophages and dendritic cells using blood sera from 3 individuals previously identified with liver toxicity (#975: male, 70-year-old; #1028: female, 65-year-old; #1037: female, 41-year-old) and 3 individuals without toxicity at C_max_ (#1012: male, 65-year-old; #1038: male, 21-year-old; #1069: female, 20-year-old) and then cultured them in transwell cell culture condition (Figure 9A). We performed a 16-day treatment protocol with redosing using ximelagatran at C_max_, emulating long-term use in the clinic, and RNAseq analysis was done on both monocyte-derived macrophage and dendritic cell populations, as well as on individual-centric spheroids. Volcano plots showed that 31 genes were differentially expressed between TOX (−) and TOX (+) conditions in individual-centric spheroids (Figure 9B), and 172 genes were differentially expressed in monocyte-derived macrophages and dendritic cells (Figure 10A). Moreover, hierarchical clustering of the RNA-seq data revealed that samples grouped primarily according to their TOX condition, indicating a high degree of similarity in their global gene expression profiles and suggesting that the physiological condition is a major determinant of transcriptomic variation in our dataset (Figures 9C, 10B). A set of 27 up-regulated DEGs (TOX (+) versus TOX (−) condition) was identified as DILI-associated genes within macrophages and dendritic cells highlighting the critical role of innate immune responses in the pathogenesis of drug-induced liver injury (Supplementary Table S1). Among them, genes such as *CD14, CD36, CD163, and C5AR1* are canonically expressed in Kupffer cells, while others like *CD52* and *SCARB2* are enriched in dendritic cells and contribute to antigen processing and immune activation. This cell-type-specific expression pattern suggests that these myeloid populations are transcriptionally responsive to hepatotoxic stress and may actively participate in amplifying liver injury through cytokine signaling, phagocytosis, and fibrotic remodeling, and thus indicating a prominent involvement of the innate immune system in the liver’s response to ximelagatran-induced injury. Gene Ontology enrichment analysis of the 27 genes upregulated in the TOX (+) condition, predominantly expressed in macrophages and dendritic cells, revealed significant enrichment in biological processes including response to stimulus, regulation of cell migration, response to oxygen-containing compounds, and response to external stimulus (Figure 10C). These findings suggest that macrophages and dendritic cells from TOX (+) condition are transcriptionally activated in response to microenvironmental stress caused by ximelagatran, and they potentially contributed to inflammation and tissue remodeling during DILI. Because there is strong evidence that polymorphisms on HLA-DRB1 and HLA-DQA1 genes are associated with ximelagatran-mediated DILI, we performed a STRING analysis on the 172 genes that were up- and downregulated in macrophages and dendritic cells upon ximelagatran treatment. We found that HLA-DRB1 and HLA-DQA1 genes were associated with the highest confidence score with VIM, a gene that was upregulated in TOX(+) condition upon ximelagatran treatment (Figure 10D). Vimentin is a cytoskeletal protein traditionally associated with cellular structure that plays critical roles in immune cell function. In macrophages and dendritic cells, vimentin is involved in migration, inflammatory signaling, and antigen presentation. Its upregulation in the TOX (+) condition may reflect enhanced activation and mobilization of these innate immune cells in response to ximelagatran-induced stress, supporting its relevance in the early events of DILI pathogenesis.

### Exploratory case studies showing the robustness of individual-centric spheroid system to predict DILI of breast cancer patients under ribociclib regimen

**FIGURE 11:**
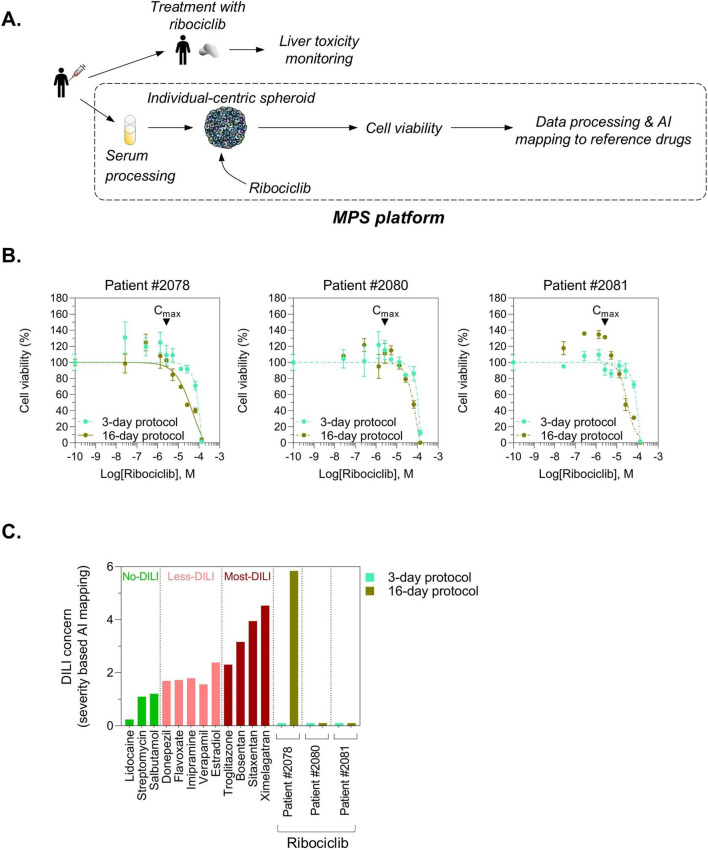
Exploratory case studies demonstrating ribociclib-induced DILI prediction via a human serum-derived educated spheroid system-based approach. **(A)** Workflow. **(B)** Nonlinear regression curves showing ribociclib dose–response in liver spheroids generated with blood serum from ER+/HER2− breast cancer patients, comparing 3-day and 16-day treatment protocols. Cell viability was measured using CellTiterGlo and results are shown as percentage ±s. e.m. of viable cells. Each condition was performed in triplicate. **(C)** *In vitro* results were mapped to reference standards using a proprietary AI-driven model incorporating a severity metric, which successfully identified a breast cancer patient who developed ribociclib-induced toxicity, in alignment with clinical observations.

Ribociclib (Kisqali®) is a targeted therapy drug that has shown significant overall survival benefit in advanced breast cancer estrogen-receptor positive (ER positive) and human epidermal growth factor receptor 2 (HER2 negative) patients (Neven et al., 2023). Preclinical studies with repeated-dose toxicity in rats and dogs have shown some hepatic alterations including cholestasis, which however were fully reversible after a 4-week recovery period off treatment (Shah et al., 2018). Interestingly, while preclinical studies demonstrated reversible hepatobiliary effects, clinical data reported of idiosyncratic and sometimes persistent liver enzyme elevations highlight the necessity for predictive tools to identify patients at risk for ribociclib-induced hepatotoxicity before starting therapy (Schaeffer et al., 2023; Atay et al., 2024). We performed 3 predictive case studies using blood sera from patients with advanced breast cancer ER+/HER-. Blood sampling was done before patients undergo treatment regimen with ribociclib. We generated individual-centric spheroids for each patient and then treated them with ribociclib using a 3-day and a 16-day protocol (Figure 11A). No liver toxicity was observed following a 3-day treatment with ribociclib, as indicated by the absence of detectable cell death at therapeutic concentrations up to the reported C_max_ value in both patients, suggesting that ribociclib does not induce acute hepatotoxicity under short-term exposure conditions (Figure 11B). However, under long-term exposure conditions (16-day protocol), patient #2078 exhibited evidence of cell death at concentrations below the therapeutic C_max_, indicating potential hepatotoxicity at clinically relevant doses. In contrast, no liver toxicity was observed in patients #2080 and #2081 under the same conditions, suggesting inter-individual variability in susceptibility to ribociclib-induced hepatotoxicity (Figure 11B). To facilitate clearer visualization of ribociclib’s potential for DILI, we applied a proprietary AI-driven mapping algorithm that compares ribociclib to reference compounds using a quantitative severity metric. This approach revealed that only patient #2078 is predisposed to ribociclib-mediated DILI at clinically relevant concentrations (Figure 11C). By comparing our *in vitro* predictive data to clinical liver toxicity monitoring in patients undergoing ribociclib treatment, we found that patient #2078 experienced grade 3 liver toxicity approximately 4 months after starting therapy, while patients #2080 and #2081 exhibited no hepatotoxic effects. These observations validate the effectiveness of our DILI risk prediction platform in identifying individual susceptibility to ribociclib-induced liver injury. While limited by a small cohort of three breast cancer patients, this study is the first to employ a human serum-derived educated spheroid system prospectively for predicting the clinical risk of DILI. The alignment between our *in vitro* predictive data and clinical observations supports further investigation and justifies larger prospective studies aimed at robustly validating the accuracy and clinical relevance of our DILI risk prediction platform.

## Discussion

In the present work, we demonstrated that individual-centric spheroids are reliable models to perform clinical trial-in-a-dish approaches to categorize less-DILI concern from most-DILI concern drugs, and importantly, to determine whether a compound causes majorly dose-dependent or dose-independent DILI. Thus, our approach supports precise drug risk classification and human stratification. Moreover, we showed that thanks to the flexibility of our technology, we could identify the mechanism of ximelagatran-mediated iDILI offering a way to mitigate the risk in individuals with iDILI predisposition. Finally, we demonstrated the robustness of our human serum-derived educated spheroid system in predicting clinical DILI through the first prospective case studies, as no previous preclinical work has confirmed reliability in detecting true DILI via prospective case analysis.

To date, advanced MPS technologies utilizing human primary tissues have aimed to predict DILI, generally providing binary (positive/negative) outcomes. Importantly, these models have not been prospectively validated through case studies to demonstrate their actual clinical predictive capability. While prospective case studies are essential to validate an MPS’s clinical predictive power for DILI, using primary liver cells in this context is not ethically or practically feasible, given the risks associated with pre-treatment liver biopsies. Our innovative approach addresses this challenge by enabling accurate DILI risk prediction through a minimally invasive method, requiring only a standard blood sample before treatment initiation. In our study, blood samples were collected prior to the patients undergoing any clinical treatment. We recognize that the number of case studies is small. However, this is the first demonstration of a human serum-derived educated spheroid system being used to prospectively confirm its predictive accuracy, which strengthens our confidence in its robustness. The current results are promising proof-of-concept, but further investigation is needed before considering clinical application. Ongoing validation efforts are focused on establishing our human serum-derived educated spheroid system as a companion diagnostic tool for predicting ribociclib-induced DILI in a large prospective cohort of breast cancer patients, in partnership with clinicians from Centre Antoine LACASSAGNE.

An increasing number of publications now document the successful conversion of hepatoma cells into metabolically functional hepatocyte-like cells. It has been shown that reprogramming HCC cells with nuclear factors such as HNF1A, HNF4A and FOXA3, can convert these cancerous cells to hepatocyte-like cells (Cheng et al., 2019). Moreover, Pramfalk and colleagues, as well as Steenbergen and colleagues reported that culturing human hepatoma cells in adult human serum can provoke a differentiation of these cells restoring hepatocyte specific functions (Pramfalk et al., 2016; Steenbergen et al., 2018). Our results are in line with these observations as we demonstrated that HepG2 line cultured in human serum at physiological proportions lost their malignant phenotype with growth arrest and reduction of the expression of Warburg genes as well as increase expression of OXPHOS genes such as COX1 and SIRT1 suggesting a shift of aerobic glycolysis towards oxidative phosphorylation. Expression of CYP family members was enhanced too. We hypothesize that this conversion of HepG2 line to hepatocyte-like cells was caused by factors such as cytokines or chemokines in the serum stimulating the expression of liver-specific transcription factors including CEBPA and CEBPB. For instance, it has been shown that extracellular signals such as IL-1, TNFα, CSF, and IL-6 can stimulate the expression of CEBPA/B (Zhang and Friedman, 2011; Ren et al., 2023). Further analyses are required to identify these serum’s factors and to confirm the role of CEBPA/B in the conversion process.

When using primary human hepatocytes to assess DILI, it is common to pool cells from multiple donors due to the limited number of viable hepatocytes that can be isolated from each individual (Faccioli et al., 2021). However, this pooling approach diminishes the ability of the model to capture interindividual variability in responses, and therefore potentially obscuring donor-specific susceptibilities to hepatotoxic drugs. Consequently, a pooling methodology can lead to inaccurate prediction of DILI risk as observed by our pooled data (data not shown). Indeed, analysis of pooling data from a 50-donor cohort revealed that donepezil and sitaxentan are non-hepatotoxic at therapeutic doses although these drugs are categorized by FDA as less-DILI concern and most-DILI concern drugs, respectively. However, when each subject from the cohort was analyzed individually, we can identify a number of subjects with dose-dependent and with dose-independent DILI (Figure 6), and importantly, we identified based on the incidence and the severity that donepezil and sitaxentan are categorized as less-DILI concern and most-DILI concern compounds, respectively (Figure 8). Our data challenges the practice of pooling primary cells for DILI risk assessment, as this approach may obscure inter-individual variability and mask susceptibility to liver injury. To boost the translational impact of our platform, we seek pharma partners to expand compound testing and build an AI model for predicting DILI risk in new drug candidates.

While primary hepatocyte cultures are the gold standard for DILI testing, their data often poorly translate to human outcomes due to dysregulated CYP expression post-isolation. *In vitro*, CYP enzymes may be overexpressed without *in vivo* regulatory controls, leading to misleading toxicity signals. It has been reported that the expression of several CYPs involved in the bioactivation of acetaminophen was found to be lower in PHHs co-cultured with non-parenchymal cells (NPCs) compared to PHHs cultured alone, suggesting that NPCs may modulate CYP expression similarly to what occurs in human livers(Bell et al., 2020). CYP expression in the human liver is tightly regulated by hormones, cytokines, exosomes, and other blood-borne factors. In response to drugs, CYPs are upregulated via nuclear receptors like PXR, CAR, and AhR to boost metabolic capacity. Isolated liver cells lack these systemic cues, often resulting in non-physiological CYP profiles that can undermine the predictive accuracy of *in vitro* models for human drug metabolism and toxicity. Further investigations combining primary human liver cells with autologous human blood serum are needed to explore this hypothesis and enhance the physiological relevance and predictive reliability of *in vitro* primary liver cell-based models.

Although our serum-educated HepG2-based system reproduces several features of primary hepatocytes and captures donor-specific responses, important limitations remain. HepG2 cells are transformed and exhibit reduced metabolic competency and attenuated stress responses compared with primary human hepatocytes, which may limit their ability to fully reflect multi-factorial mechanisms such as the second-hit processes implicated in idiosyncratic DILI. The inclusion of TWNT-1 cells and THP-1-derived macrophages and dendritic cells partly restores inflammatory and stress-related signaling, but it cannot completely overcome the intrinsic constraints of HepG2 cells. Nonetheless, the system benefits from its reproducibility, accessibility, and alignment with previous work demonstrating its capacity to detect DILI-associated signatures. Future extensions, including the integration of additional stressors or more stress-competent hepatic models, may further enhance their ability to capture complex liver injury mechanisms. Moreover, we recognize that HepG2 cells have lower metabolic activity than primary hepatocytes. However, our model is not limited to HepG2 cells. It also incorporates TWNT-1 cells and THP-1-derived macrophages and dendritic cells, all educated with the same donor serum. These additional cell populations modulate HepG2 responses through paracrine and inflammatory signaling, creating a more physiologically relevant microenvironment. As a result, a meaningful comparison with a primary system would require not only primary hepatocytes, but also primary stellate cells and Kupffer cells derived from the same donor, along with donor-matched serum to maintain comparable conditions. Acquiring all of these primary cell types and sufficient serum from a single donor is extremely challenging due to limited tissue availability, variability in cell yield and viability, and practical and ethical constraints. While such a fully donor-matched comparison would be scientifically valuable and remains an interesting future direction, it is currently difficult to achieve in practice.

A limitation of the present study is the use of serum with inherent variability, which may impact the reproducibility of the findings across experiments using cells from different donors. Differences in serum composition can influence cellular responses and contribute to donor-dependent variability in experimental outcomes. Furthermore, these effects may be more pronounced when translating the results to primary cell models, which typically display greater heterogeneity and sensitivity to culture conditions. Accordingly, the findings should be interpreted with these limitations in mind, and future studies employing defined culture conditions and primary cells will be important to further validate and extend the conclusions.

DILI is a complex, multi-parametric process, and viability alone cannot fully represent its diverse manifestations. We selected ATP-based viability as our primary endpoint because it offers a rapid, cost-effective, and highly reproducible readout that is well suited for systematic comparisons across multiple drugs and donor-derived spheroids. However, our human serum-educated spheroid platform is inherently compatible with a wide range of additional mechanistic endpoints. In future work, we plan to incorporate complementary measures such as enzyme leakage, oxidative stress markers, and inflammatory cytokine profiles. The inclusion of these multi-parametric readouts will enable the detection of earlier or more nuanced hepatotoxic responses, improve the resolution of severity assessments, and further enhance the translational relevance of the model.

In this study, all tested compounds were correctly classified according to the FDA-defined DILI categories, yielding a preliminary 100% concordance for this specific dataset. To our knowledge, no current *in vitro* model has demonstrated the capability to distinguish compounds not only as DILI-positive or -negative but also across graded categories such as no-, less-, and most-DILI. However, meaningful estimates of sensitivity and specificity require evaluation across a substantially larger and more diverse compound panel, typically, 50–100 drugs spanning a broad range of DILI risks are needed to generate robust performance metrics. Thus, while our results offer a proof-of-concept for graded DILI prediction, larger-scale studies will be essential to rigorously establish sensitivity and specificity and to benchmark our platform against existing approaches. *In vitro* models are typically employed to assess DILI risk following either short-term or long-term exposure. However, these exposure durations are rarely evaluated systematically within the same experimental framework. As a result, this fragmented approach may fail to capture the full spectrum of a drug’s hepatotoxic potential, potentially overlooking key aspects of its true DILI risk. We observed that ximelagatran exhibits a shift in its DILI profile over time. Indeed, it showed a time-dependent shift in hepatotoxic potential, transitioning from a low risk during short-term use to a high risk of DILI with prolonged exposure. Thus, based on our data, we believe that for accurate determination of a new drug’s DILI risk, including its placement on the hepatotoxicity spectrum, we absolutely need comprehensive assessment under both acute and chronic treatment conditions. Our technology offers that possibility and consequently will be a valuable platform for pharmaceutical companies and regulators to de-risk DILI. The intended context of use of our system is to support individualized DILI risk prediction rather than broad preclinical screening across large drug libraries. This aligns with regulatory expectations that evidentiary strength be demonstrated within the specific population and exposure conditions relevant to clinical use. At this stage, the model represents a proof-of-concept framework, and further work, including expansion of the reference drug set and larger prospective studies, will be required to establish sensitivity and specificity for future regulatory qualification.

Presently, the mechanism responsible for ximelagatran-mediated DILI remains unclear. There is strong evidence that some HLA polymorphisms such as DRB1*07:01 and DQA1*02:01, are associated with ximelagatran-mediated DILI (Keisu and Andersson, 2010). Certain HLA alleles are associated with higher or lower baseline levels of serum cytokines as they may regulate macrophages’ secretion activity, influence their function and behavior (Ambarus et al., 2018; van Drongelen et al., 2021; Prapas and Anagnostouli, 2024). For instance, they can influence the polarization of macrophages into either pro-inflammatory or anti-inflammatory phenotypes. Thus, macrophages which express high levels of HLA-DR, secrete pro-inflammatory cytokines such as TNFα, IL-1β, and IL-12. In contrast, macrophages which have reduced HLA-DR expression, secrete anti-inflammatory cytokines such as IL-10 and TGF-β. While vimentin does not directly regulate transcription of HLA class II genes such as DRB1 and DQA1, it plays a supportive role in the antigen-presenting function of macrophages and dendritic cells (Yu et al., 2018; Thalla et al., 2022). Through its involvement in endosomal trafficking, cytoskeletal organization, and inflammatory signaling, vimentin may facilitate the proper expression, loading, and surface presentation of HLA class II molecules. Its upregulation in activated innate immune cells may therefore reflect or contribute to enhanced antigen presentation capacity during immune responses, including DILI. Additional studies are needed to elucidate the specific role of vimentin in macrophage and dendritic cell responses during ximelagatran-mediated DILI.

Although we do not have direct mechanistic evidence to explain why chronic administration of ximelagatran leads to increased hepatotoxicity compared with short-term treatment, our data demonstrate that the human serum-derived educated spheroid system can capture ximelagatran-mediated DILI under chronic exposure conditions, but not under acute treatment, closely mirroring the clinical observations in humans. One potential explanation is that chronic exposure allows cumulative stress and interactions between the different cell types in our system, including HepG2, stellate, and immune cells, modulated by donor serum, which may amplify susceptibility to DILI, whereas acute exposure does not sufficiently engage these mechanisms.

The use of AI-driven approaches to predict DILI risk remains challenging due to the heterogeneity of input data. These data are often generated under varying experimental conditions such as differences in cell culture protocols, exposure durations, and data collection methods, resulting in inconsistencies that undermine data quality. Consequently, the reliability of AI predictions is compromised, as low-quality or non-standardized inputs can lead to misleading conclusions about a drug’s true hepatotoxic potential. Mohammed and colleagues have analyzed the relationship between 6 data quality dimensions and concluded that low-quality data can significantly impair model performance, leading to unreliable predictions (Mohammed et al., 2025). Moreover, this issue is further amplified when large volumes of data are required to train AI models. The need for high-throughput data generation often leads to greater variability in experimental conditions, further compromising data consistency and quality. As a result, the scalability of AI approaches for DILI prediction can inadvertently amplify errors, increasing the risk of drawing inaccurate conclusions about a compound’s hepatotoxicity. To address these limitations, we developed a robust and standardized *in vitro* system that captures human population-level biological diversity. The uniqueness of our human serum-derived educated spheroid system lies in its ability to generate data from a large cohort of either randomly selected or specifically tailored individuals, i.e., the presence of specific diseases or known vulnerabilities including smoking status, alcohol consumption, obesity, and other relevant health conditions, all processed using a standardized methodology. This uniform approach to sample preparation enables the generation of consistent, high-quality datasets suitable for powering AI-driven models, thereby enhancing the reliability of DILI risk prediction. We believe that integrating data generated from our standardized *in vitro* system with clinical data and mechanistic insights will significantly enhance the predictive power of AI models. This multi-dimensional approach has the potential to improve the accuracy and reliability of DILI risk assessment for new drug candidates. While we are unable to provide detailed technical information on the AI algorithm due to confidentiality, we can clarify that it evaluates toxicity based on therapeutic doses rather than supra-physiological concentrations. This design ensures that the analysis reflects clinically relevant exposures and avoids overestimating toxicity that might occur at unrealistically high doses. By focusing on therapeutic ranges, the AI provides a more accurate assessment of DILI incidence and severity, which underlies the classification of compounds as DILI-positive or DILI-negative in our study. We are now seeking further partnerships with industry stakeholders and regulatory agencies to advance the development of our AI-driven *in vitro* platform. These collaborations aim to support more effective DILI de-risking at the preclinical stage and facilitate the adoption of predictive, data-driven approaches in drug safety assessment.

Recent efforts to improve *in vitro* liver models have underscored the importance of three-dimensional (3D) hepatocyte cultures, which better recapitulate *in vivo* architecture and function than traditional two-dimensional systems and are increasingly applied for drug metabolism, disposition, and toxicity studies. A notable example is the development of chemically defined, animal serum-free 3D primary human hepatocyte spheroid cultures, which eliminate fetal bovine serum, a variable, poorly characterized animal product, and demonstrate comparable viability and cytochrome P450 function, thus improving reproducibility and ethical compliance in long-term studies (Mickols et al., 2025). We believe that our human serum-derived educated spheroid system will be complementary to these serum-free systems, potentially enhancing translational interpretation and reducing both false positive and false negative DILI predictions.

Finally, an important future direction for our work is the inclusion of serum samples from diverse ethnic and vulnerable populations, as large registry and cohort studies have shown that DILI severity can differ across populations, presumably because of individual-specific predispositions, environmental factors, cultural lifestyle, and drug-use patterns. Our human serum-derived educated spheroid system is uniquely positioned to capture such population variability because it directly integrates donor-specific serum, unlike primary hepatocyte-based models, which are constrained by the limited availability of primary liver cells from diverse populations. Expanding the system to include serum from additional groups will therefore provide an opportunity to better understand population-specific DILI susceptibility and further enhance the translational relevance of the model.

## Data Availability

The datasets presented in this article are not readily available because they are the exclusive property of PredictCan Biotechnologies. Requests to access the datasets should be directed to ht.duong@predictcan.com.
